# Is there an optimal inter-delivery interval in women who underwent trial of labor after cesarean delivery (TOLAC)?

**DOI:** 10.1186/s12978-021-01319-0

**Published:** 2022-01-20

**Authors:** Jiaming Rao, Dazhi Fan, Huiting Ma, Dongxin Lin, Huishan Zhang, Zixing Zhou, Pengsheng Li, Gengdong Chen, Demei Lu, Yan Liu, Zhaoxia Wu, Jieyun He, Xinjuan Liu, BingJie Peng, Xiaoling Guo, Zhengping Liu

**Affiliations:** 1 Foshan Fetal Medicine Institute, Affiliated Foshan Maternity & Child Healthcare Hospital, Southern Medical University (Foshan Maternity & Child Healthcare Hospital), Renminxi Road 11, Foshan, 528000 Guangdong China; 2 Department of Obstetrics, Affiliated Foshan Maternity & Child Healthcare Hospital, Southern Medical University (Foshan Maternity & Child Healthcare Hospital), Renminxi Road 11, Foshan, 528000 Guangdong China; 3 Department of Obstetrics, Nanhai Maternity & Child Healthcare Hospital, Foshan, 528200 Guangdong China; 4 Department of Obstetrics, Shunde Maternity & Child Healthcare Hospital, Foshan, 528300 Guangdong China; 5 Department of Obstetrics, The People’s Hospital of Gaoming, Foshan, 528500 Guangdong China; 6 Department of Obstetrics, Sanshui Maternity & Child Healthcare Hospital, Foshan, 528100 Guangdong China

**Keywords:** Trial of labor after cesarean delivery, Inter-delivery interval, Birth spacing, Uterine rupture, Maternal and neonatal outcomes

## Abstract

**Background:**

Inter-delivery interval (IDI) has been proven to be a factor associated with adverse maternal and neonatal outcomes. However, the optimal IDI in trial of labor after cesarean delivery (TOLAC) remains unclear. We aimed to investigate the association between IDI and major maternal and neonatal outcomes in women who underwent TOLAC.

**Methods:**

A multicenter, retrospective cohort study including five hospitals was conducted between January 2018 and December 2019 in Foshan, China. This study included 1080 pregnant women with one or two cesarean deliveries who attempted a TOLAC. Data on maternal and neonatal outcomes were collected from the electronic record system. Maternal and neonatal outcomes in different groups of IDI were compared by univariate and multivariable analyses.

**Results:**

A short IDI of < 24 months did not show a statistically significant association with uterine rupture in the univariate analysis (*P* = 0.668). In multivariable analysis, the incidences of postpartum hemorrhage (OR 19.6, 95% CI:4.4–90.9, *P* < 0.05), preterm birth (OR 5.5, 95% CI:1.5–21.3, *P* < 0.05), and low birth weight (OR 3.5, 95% CI:1.2–10.3, *P* < 0.05) were significantly increased in women with an IDI of < 24 months than in those with a normal interval (24–59 months). Infection morbidity (OR 1.8, 95% CI:1.4–7.9, *P* < 0.05), transfusion (OR 7.4, 95% CI:1.4–40.0, *P* < 0.05), and neonatal unit admission (OR 2.6, 95% CI:1.4–5.0, *P* < 0.05) were significantly increased in women with an IDI of 120 months or more than in those with a normal interval. Postpartum hemorrhage (*P* = 0.062) had a trend similar to that of a significant IDI of 120 months or more. We found no statistically significant difference in maternal and neonatal outcomes between 24–59 months and 60–119 months.

**Conclusions:**

An IDI of less than 24 months or 120 months or more increased the risk of major maternal and neonatal outcomes. We recommend that the optimal interval for women who underwent TOLAC should be 24 to 119 months.

**Supplementary Information:**

The online version contains supplementary material available at 10.1186/s12978-021-01319-0.

## Plain Language summary

An inter-delivery interval (IDI) that is too short or too long increases the risk of adverse maternal and neonatal outcomes. However, the optimal IDI for trial of labor after cesarean delivery (TOLAC) remains unclear. We performed a multicenter, electronic medical record-based, retrospective cohort study that included 1080 pregnant women who had one or two cesarean deliveries and underwent TOLAC. Data on maternal and neonatal outcomes were collected from the electronic record system. In multivariable analysis, the incidences of postpartum hemorrhage, preterm birth, and low birth weight were significantly increased in women with an IDI of < 24 months than in those with a normal interval (24–59 months). Infections, transfusion, and neonatal unit admission were significantly increased in women with an IDI of ≥ 120 months than in those with a normal interval. In conclusion, we found that an IDI < 24 months or ≥ 120 months increased the risk of major maternal and neonatal outcomes. We recommend that the optimal interval for women who underwent TOLAC should be 24 to 119 months.

**Supplementary Information:**

The online version contains supplementary material available at 10.1186/s12978-021-01319-0.

## Background

An inter-delivery interval (IDI) that is too short or too long increases the risk of adverse maternal and neonatal outcomes [1–4]. However, the optimal IDI in trial of labor after cesarean delivery (TOLAC) remains unclear [5–11].

Studies on TOLAC IDI had limited study designs and small sample sizes [5–11]. Some studies [5–8] reported that short birth intervals increased adverse outcomes, such as uterine rupture and decreased success rates of vaginal birth after cesarean (VBAC). On the contrary, other studies [9–11] suggested that there was no association between IDI and uterine rupture. Current guidelines are based on the occurrence of serious complications, such as uterine rupture, to determine the IDI in women undergoing TOLAC [12–14]. The American College of Obstetricians and Gynecologists (ACOG) suggested that an IDI of < 19 months reduced the success rate of VBAC [5, 12]. Furthermore, the Society of Obstetricians and Gynaecologists of Canada proposed that women with an IDI of < 18 months had an increased risk of uterine rupture when undergoing TOLAC [13]. Moreover, China’s 2016 edition of TOLAC expert consensus [14] suggested that the TOLAC IDI should be > 18 months.

We believe that the optimal IDI must include several factors in women undergoing TOLAC. First, the incidence of serious complications, such as uterine rupture, does not increase. In other words, prior incisions recover well and tolerate the increased uterine volume in the current pregnancy. Second, women are within a relative young age for childbirth. Third, other maternal and child complications do not increase significantly. Thus, the delivery method should be based not only on the risk of uterine rupture, but also on the other potential adverse maternal and neonatal outcomes. The World Health Organization recommends that women should wait for at least 24 months after childbirth before the conception of the next child to reduce the incidence of complications [15]. This recommendation is longer than that stated in the current TOLAC guidelines [12–14]. In addition, clinicians are relatively more conservative about IDI, thereby providing recommendations that are longer than those in existing guidelines.

Therefore, the present study aimed to determine the optimal IDI for women undergoing TOLAC, to investigate the relationship between IDI and maternal and neonatal outcomes, and to analyze the relationship between an IDI of ≥ 120 months and delivery outcomes, considering that this relationship has not been analyzed in previous studies.

## Methods

### Study population

We performed a multicenter, electronic medical record-based, retrospective cohort study that included 1080 pregnant women who had one or two cesarean deliveries and underwent TOLAC between January 2018 and December 2019. Foshan is divided in five districts, namely Chancheng, Nanhai, Shunde, Sanshui, and Gaoming, covering an area of 3797.72 km^2^. A total of five hospitals in the Foshan, provinces of Guangdong participated in the study. Of the five, three were tertiary hospitals and two were secondary. The hospitals were Foshan Maternity & Child Healthcare Hospital, Nanhai Maternity & Child Healthcare Hospital, Shunde Maternity & Child Healthcare Hospital, Sanshui Maternity & Child Healthcare Hospital, and The People’s Hospital of Gaoming. These hospitals were from five administrative districts in Foshan City. In these hospitals, which cater to a population of more than 8 million, a total of 36,000 births were recorded in 2020. Data were collected from these hospitals by trained research obstetricians and nurses using uniformed, structured, and closed-ended data forms based on electronic medical records.

The inclusion criteria were as follows: (1) women aged 18–40 years who decided to undergo TOLAC; (2) one or two previous cesarean deliveries with a lower uterine transverse incision but without other uterine surgical scars; and (3) cephalic presentation and absence of previous or new indications for cesarean delivery. The exclusion criteria were as follows: (1) previous classical cesarean section with a T-shaped incision; (2) uterine incision caused by surgery other than a cesarean section., uterine rupture, or dehiscence; (3) multiple pregnancies, breech presentation, or threatening uterine rupture or dehiscence; and (4) other factors not suitable for vaginal delivery.

This study was approved by the Human Subjects Committee of the Affiliated Foshan Maternity & Child Healthcare Hospital, Southern Medical University (Approval number FSFY-MEC-2018-021). To ensure patient privacy, our data did not include the patients’ name, phone number, home address, and other sensitive information.

### Data collection and definitions

We calculated the gestational age based on the last menstrual period and actual delivery dates in the medical records. Uterine rupture was defined as a separation of the uterine scar, which was determined during laparotomy. Uterine rupture was commonly preceded by symptoms, such as abnormal fetal heart rate pattern, maternal signs or symptoms of acute blood loss, or hematuria. Low birth weight (LBW) was defined as a birth weight < 2500 g. Body mass index (BMI) was calculated using the height and weight of the baby at the first prenatal visit or of the fetus before delivery (BMI = weight (in kg)/height^2^ (in m)). Gestational diabetes mellitus was defined as fasting plasma glucose  ≥ 5.1 mmol/L (92 mg/dL) or a 1-h plasma glucose  ≥ 10.0 mmol/L (180 mg/dL) or a 2-h plasma glucose ≥ 8.5 mmol/L (153 mg/dL) [16]. Preeclampsia was defined as a systolic BP of ≥ 140 mmHg and a diastolic BP of ≥ 90 mmHg after 20 weeks of gestation in a previously normotensive woman with 24-h urine collection of ≥ 300 mg [17]. BP was recorded on two separate occasions, at > 4-h apart for preeclampsia. Infections morbidity was defined by symptoms of fever, infection, endometritis, and chorioamnionitis. Postpartum hemorrhage was defined by ACOG as a blood loss of more than 500 mL for vaginal deliveries and a volume of more than 1000 mL for cesarean deliveries [18].

The current literature uses two expressions: inter-pregnancy interval (IPI) or IDI to define the interval, which is easily misunderstood and difficult to standardize. This study recommends the use of IDI, which is defined as the date of the last cesarean delivery from the date of the delivery of the current pregnancy in women undergoing TOLAC. IDI is calculated as the interval between live births, excluding miscarriages and fetal deaths of the last cesarean delivery. We believe that the conception of the current pregnancy is a date that has to be calculated rather than a definite date, whereas the period between the delivery of the previous birth and the delivery of the current pregnancy could be determined. Based on prior research and our data, we divided the IDI into < 24, 24–59, 60–119, and ≥ 120 months between the prior cesarean delivery date and the subsequent delivery date. IDI between 24 to 59 months is defined as normal interval and reference interval.

The sample size was calculated using PASS 15.0.5 (Kaysville, Utah, USA). We defined the main maternal and infant outcomes based on pre-experimental results. The postpartum hemorrhage rate (p1) for 24–59 months was expected to be 1%, the postpartum bleeding rate (p2) for IDI < 24 months was expected to be 5%, and the sample size was 754 according to the design of Tests for two scales, based on power = 0.9 and alpha = 0.05. The final sample size for this study was 1080.

### Statistical analyses

Categorical variables are reported as numbers and percentages, whereas continuous variables are presented as mean ± standard deviation (SD). Statistical significance was calculated using Pearson’s Chi-squared test or Fisher’s exact test for differences in qualitative variables and Student’s t-test or the Kruskal–Wallis rank test for differences in continuous variables.

Differences in the number and proportion of IDI were calculated, including descriptive characteristics (maternal age and maternal nationality), obstetric characteristics (parity, number of cesarean deliveries, any prior vaginal delivery, BMI at the first prenatal visit, BMI at TOLAC delivery, estimated fetal weight at TOLAC delivery, estimated fetal head circumference, lower uterine segment thickness, cervical dilatation on admission at TOLAC delivery, cervical effacement on admission at TOLAC delivery, cervical score, and pre-existing medical condition), maternal outcomes (success rate of TOLAC, infection morbidity, postpartum hemorrhage, transfusion, uterine rupture, hysterectomy, and maternal death), and neonatal outcomes (premature birth, LBW, APGAR score < 5 at 1 min, APGAR score < 5 at 5 min, stillbirth, and neonatal unit admission).

Multinomial logistic regression models were used to estimate the likelihood of maternal outcomes using the four-category IDI, which was adjusted for maternal nationality, prior cesarean number, gestational age, maternal age, maternal BMI at delivery, parity, GDM and preeclampsia. In stepwise options, we used the likelihood ratio test with an entry and removal probability of 0.1. All unadjusted variables (*P* < 0.1) were inputted into multivariable logistic models as outcome variables or as adjustment variables according to our research purpose. Statistical analyses were performed using SPSS, version 24.0 (IBM Corp., Armonk, NY, USA).

## Results

The final analysis included 1080 patients; of these patients, 868 (80.4%) had a VBAC, whereas the remaining 212 (19.6%) had a failed TOLAC. Descriptive and obstetric characteristics of women who attempted TOLAC in these two groups are shown in Additional file 1: Table S1. There were 28 (2.6%), 395 (36.6%), 521 (48.2%), and 112 (10.4%) women who had IDIs of < 24, 24–59, 60–119, and ≥ 120 months, respectively. The remaining 24 (2.2%) women had missing IDI data. There were eight cases of uterine rupture, with a rupture rate of 0.7% (8/1080) in all participants.

Women with a short IDI (< 24 months) were more likely to have higher parity and preeclampsia, more than one prior cesarean delivery, lower BMI at delivery, and lower estimated fetal weight at TOLAC delivery than those with a normal interval (between 24 and 59 months). Women with a long IDI (≥ 120 months) were more likely to have higher parity, preeclampsia, BMI at the first prenatal visit, BMI at delivery, and proportion of any prior vaginal delivery and a lower estimated fetal weight at TOLAC delivery than those with a normal interval. Women with a short (< 24 months) or long (≥ 120 months) IDI more commonly had preeclampsia than those with a normal interval (Table 1).

**Table 1:** Descriptive and obstetric characteristics of women who attempted vaginal birth after cesarean delivery by inter-delivery interval length

Variable	Inter-delivery interval (completed month)				P-value
	Less than 24 (n = 28)	24–59 (n = 395)	60–119 (n = 521)	120 or more (n = 112)	
Maternal age (y, s.d.)	31.0 ± 3.3	31.4 ± 4.2	32.1 ± 4.2	32.2 ± 4.2	0.061
Maternal Nationality (%)					0.730
Han	28 (100.0)	384 (97.2)	510 (97.9)	110 (98.2)	
Other minorities	0	11 (2.8)	11 (2.1)	2 (1.8)	
Parity (times, s.d.)	1.4 ± 1.0	1.2 ± 0.4	1.3 ± 0.5	1.5 ± 0.7	< 0.001
Number of CD (%)					0.034
1	27 (96.4)	393 (99.5)	520 (99.7)	112 (100.0)	
2	1 (3.6)	2 (0.5)	1 (0.2)	0	
Any prior vaginal delivery (%)	4 (14.3)	56 (14.2)	124 (23.8)	43 (38.4)	< 0.001
BMI at the first prenatal visit (kg/m^2 ^s.d.)	21.7 ± 3.2	21.3 ± 2.8	21.6 ± 3.1	22.4 ± 3.2	0.007
BMI at TOLAC delivery (kg/m^2^, s.d.)	25.7 ± 3.5	25.9 ± 2.9	26.5 ± 3.2	26.8 ± 3.2	0.003
Estimated fetal weight at TOLAC delivery (g)	2868 ± 593	3043 ± 420	3049 ± 471	2914 ± 513	0.033
Estimated fetal head circumference (mm, s.d.)	311 ± 31	333 ± 233	328 ± 186	307 ± 57	0.694
Lower uterine segment thickness (mm, s.d.)	2.9 ± 0.3	2.7 ± 0.6	2.6 ± 0.6	2.8 ± 0.5	0.099
Cervical dilatation on admission at TOLAC delivery (5–6 cm)	4 (19.0)	56 (17.4)	80 (20.9)	17 (23.6)	0.878
Cervical effacement on admission at TOLAC delivery (80–100%)	17 (81.0)	253 (78.6)	309 (80.3)	56 (77.8)	0.891
Cervical score (score, s.d.)	7.9 ± 2.3	7.7 ± 2.7	8.1 ± 2.7	8.1 ± 2.9	0.215
Pre-existing medical condition (%)					
GDM	5 (17.9)	65 (16.5)	114 (21.9)	27 (24.1)	0.138
Preeclampsia	4 (14.3)	6 (1.5)	9 (1.7)	11 (9.8)	< 0.001

The univariable analysis was used to determine that women with a short IDI (< 24 months) were more likely to have a higher proportion of postpartum hemorrhage, preterm birth (PB), LBW, and neonatal unit admission than those with a normal interval. Women with a long IDI (≥ 120 months) were more likely to have a higher proportion of postpartum hemorrhage, PB, LBW, APGAR score < 5 at 5 min, and antepartum stillbirth than those with a normal interval (Table 2).

**Table 2:** Maternal and neonatal outcomes of women who attempted vaginal birth after cesarean delivery by inter-delivery interval length

Maternal and neonatal risks	Inter-delivery interval (completed month)				P
	Less than 24 (n = 28)	24–59 (n = 395)	60–119 (n = 521)	120 or more (n = 112)	
Maternal					
Successful rate of TOLAC	25 (89.3)	322 (81.5)	411 (78.9)	89 (79.5)	0.477
Infections morbidity	0	5 (1.3)	0 (1.7)	3 (2.7)	0.224
Postpartum hemorrhage	2 (7.1)	3 (0.8)	13 (2.5)	4 (3.6)	0.036
Transfusion	0	3 (0.8)	8 (1.5)	3 (2.7)	0.082
Uterine rupture	0	4 (1.0)	4 (0.8)	0	0.668
Hysterectomy	0	0	0	0	–
Maternal death	0	0	0	0	–
Neonatal					
PB (< 37 weeks)	12 (42.9)	42 (10.7)	59 (11.3)	19 (17.0)	< 0.001
LBW	8 (28.6)	31 (7.9)	43 (8.3)	15 (13.4)	0.001
APGAR < 5 at 1 min	0	2 (0.5)	2 (0.4)	3 (2.7)	0.050
APGAR < 5 at 5 min	0	0	1 (0.2)	2 (1.8)	0.014
Antepartum stillbirth	0	0	1 (0.2)	2 (1.8)	0.014
Intrapartum stillbirth	0	0	0	0	–
Neonatal unit admission	5 (17.9)	35 (9.0)	60 (11.6)	16 (14.5)	0.133

Infections morbidity: Defined as fever, infection, endometritis, and chorioamnionitis

Multinomial logistic regression analysis was used to indicate that postpartum hemorrhage (OR 19.6, 95% CI 4.4 to 90.9, *P* < 0.05), PB (OR 5.5, 95% CI 1.5 to 21.3, *P* < 0.05), and LBW (OR 3.5, 95% CI 1.2 to 10.3, *P* < 0.05) were associated with an increased risk for having a short IDI (< 24 months) than in women with a normal interval. Infection morbidity (OR 1.8, 95% CI 1.4 to 7.9, *P* < 0.05), transfusion (OR 7.4, 95% CI 1.4 to 40.0, *P* < 0.05), and neonatal unit admission (OR 2.6, 95% CI 1.4 to 5.0, *P* < 0.05) were associated with an increased risk for a long IDI (≥  120 months) than in women with a normal interval. Postpartum hemorrhage (OR 4.0, 95% CI 0.9 to 18.9, *P* = 0.062) had a trend similar to a significant IDI of ≥ 120 months (Table 3).

**Table 3:** Multinomial logistic regression analysis of maternal outcomes using the four-category inter-delivery interval

Outcome	Inter-delivery interval (completed month)			
	Less than 24	24–59	60–119	120 or more
Maternal				
Infection morbidity	–	Ref	1.3 (0.4–4.2)	1.8 (1.4–7.9)*
Postpartum hemorrhage	19.6 (4.4–90.9)*	Ref	2.2 (0.6–8.1)	4.0 (0.9–18.9)
Transfusion	–	Ref	2.2 (0.5–10.9)	7.4 (1.4–40.0)*
Neonatal				
PB (< 37 weeks)	5.5 (1.5–21.3)*	Ref	0.8 (0.4–1.6)	1.1 (0.4–2.8)
LBW	3.5 (1.2–10.3)*	Ref	0.9 (0.4–1.8)	1.4 (0.5–3.6)
Neonatal unit admission	0.7 (0.2–2.4)	Ref	1.2 (0.7–2.1)	2.6 (1.4–5.0)*

Data are expressed as adjusted odds ratios and 95% confidence intervals

Infection morbidity: Defined as fever, infection, endometritis, and chorioamnionitis

Multivariable models included the following covariates: Maternal nationality, prior cesarean number (one compared with two), gestational age, maternal age, maternal BMI at delivery, parity, GDM and preeclampsia

^*^Significant findings (P < 0.05)

## Discussion

We performed a multicenter, electronic record-based, retrospective cohort study that included 1080 pregnant women who had one or two prior cesarean deliveries and attempted TOLAC between 2018 and 2019 at five hospitals. In this multicenter study, we demonstrated that a short IDI (< 24 months) was an independent risk factor for postpartum hemorrhage, PB, and LBW in women who underwent TOLAC. We also demonstrated that a long IDI (> 120 months) was an independent risk factor for morbidity, including infections, transfusion, and neonatal unit admission in women who underwent TOLAC. However, uterine rupture was not significantly associated with short or long IDI.

Most studies and guidelines support that short IDI was associated with an increased risk of uterine rupture [7, 8, 12, 13]. Bujold et al. [7] reported that an IDI shorter than 18 months was associated with a significant increase in uterine rupture (OR 3.0, 95% CI 1.3 to 7.2); in contract, an IDI ranging from 18 to 24 months was not associated with uterine rupture. Stamilio et al. [6] analyzed 13331 women who had an IPI of < 6, 6–11, 12–17, and ≥ 60 months and found that an IPI of < 6 months was associated with an increased risk of uterine rupture (OR 2.66, 95% CI 1.21 to 5.82) and other major morbidities. IPI was inversely associated with the likelihood of a uterine scar failure during subsequent labor in a case–control study [8]. However, three other studies did not observe an association between IDI and uterine rupture [5, 10, 11]. Existing studies were contradictory to each other, which may be attributed to the inconsistencies in definition, sample sizes, and research designs. Thus, further studies are needed to clarify the correlation.

The majority of these studies did not report a relationship between IDI and other mild adverse outcomes in women undergoing TOLAC and overlooked the relationship between prolonged IDI (≥ 120 months) and adverse outcomes. Hence, we conducted this study. We found that adverse outcomes were associated not only with a short IDI, but also with a prolonged IDI (≥ 120 months). Since the rescindment of China’s single-child policy, the large increase in the number of older pregnant women provided us with the best platform to observe the relationship between long IDI (> 10 years) and adverse maternal and neonatal outcomes.

We believe that the recommendations regarding IDI in the guidelines [12–14] excessively focus on serious obstetric complications, such as uterine rupture, and give insufficient attention to other common obstetric complications, such as postpartum bleeding, infection, and PB. We believe that serious complications alone could not be the only basis for determining the optimal IDI. Therefore, we suggest that the optimal IDI of women who attempt TOLAC is 24–119 months, which could result in the best maternal and neonatal outcomes.

This study presented an optimal IDI for women who attempted TOLAC. However, because of the low number of people who underwent TOLAC with an IDI of < 24 months in this study, some existing associations may have been missed. Previous studies [5–8] reported that a short IDI was associated with an increased risk of uterine rupture. This study was not intended to overturn or refute these conclusions. Instead, this study showed that the overall incidence of uterine rupture in women who attempted TOLAC was low, even among women with short IDI who were closely monitored using currently available techniques.

The subjects of this study were appropriate candidates for TOLAC. Our team followed our exclusion criteria strictly. This study enrolled only women who met all inclusion criteria and attempted TOLAC. We did not include women with serious complications of prior cesarean delivery or twin births in the current study. In addition, the recommended scope of IDI was 24–119 months. However, specific recommendations should be customized when applied in the clinical setting. For instance, a 35-year-old pregnant woman recovers well from a cesarean delivery. Considering her age, a reasonable recommendation for IDI is 2 years apart. If a 25-year-old woman exhibits good fertility in the next decade, the recommended IDI can be extended to 119 months.

## Strengths and limitations

This was a multicenter study; the sample was a good representation of the population and could truly reflect TOLAC situation in the Foshan area, which allowed us to provide a reliable correlation between IDI and maternal and neonatal outcomes. In addition, we found an association between a long IDI (≥ 120 months) and maternal and neonatal adverse outcomes, which were rarely reported in previous studies and effectively complemented current evidences.

This study also has some limitations. First, uterine rupture is rare; thus, a large sample size is required to provide robust conclusions; Second, when studying the relationship between time variables, such as IDI and clinical outcomes, age itself is a risk factor for most diseases. As age increases, the elasticity of scar tissue formed by cesarean section decreases, and the complications of age itself increase. A magnetic resonance imaging study showed that the restoration of a lower segment hysterotomy required a total of 6–9 months [19]. However, no studies have reported the relationship between IDI and the elasticity of scar and muscle. Thus, we adjusted the maternal age in the multivariable analysis to minimize the effect of age on the results.

## Conclusions

A short (< 24 months) IDI and a long (≥ 120 months) IDI were associated with an increased risk of major maternal and neonatal outcomes. The overall incidence of uterine rupture in women who attempted TOLAC was low. We recommend that the optimal interval for women who underwent TOLAC should be 24 to 119 months to reduce the risk of major maternal and neonatal outcomes.

## Supplementary Information

**Additional file 1: Table S1.** Descriptive and obstetric characteristics of women who attempted TOLAC.

## Data Availability

Data are available upon request from the corresponding author.

## Abbreviations

TOLAC: Trial of labor after cesarean section
VBAC: Vaginal birth after cesarean section
IDI: Inter-delivery interval
ACOG: The American College of Obstetricians and Gynecologists
LBW: Low birth weight
BMI: Body mass index
PB: Preterm birth
