# Supplementary material for: Is there an optimal inter-delivery interval in women who underwent trial of labor after cesarean delivery (TOLAC)?
Source: Reprod Health. 2022 Jan 20;19:14. doi: 10.1186/s12978-021-01319-0 (PMC8772215; doi:10.1186/s12978-021-01319-0)
Supplement: Supplementary file 1 — Additional file 1: Table S1. Descriptive and obstetric characteristics of women who attempted TOLAC. [file 12978_2021_1319_MOESM1_ESM.pdf]

Table S1 Descriptive and obstetric characteristics of women who attempted TOLAC

Variable	Successful TOLAC (n=868)	Failed TOLAC (n=212)	P-value
Maternal age (y, s.d.)	31.9±6.7	33.4±9.2	0.011
Maternal Nationality (%)			0.297
Han	845(97.4)	209(98.6)	
Other minorities	23(2.6)	3(1.4)	
Parity	1.3±0.6	1.2±0.4	<0.001
Number of CS			0.322
1	864(99.5)	212(100.0)	
2	4(0.5)	0	
Any prior vaginal delivery	210(24.2)	29(13.7)	0.017
BMI at first prenatal visit (kg/m2)	21.5±3.0	21.9±3.0	0.103
BMI at delivery (kg/m2)	26.1±3.0	26.8±3.3	0.005
Time interval between last cesarean section (months)			0.550
0 – 23 months	25(2.9)	3(1.4)	
24 – 59 months	322(38.0)	73(34.9)	
60 – 119 months	411(48.5)	110(52.6)	
120 - months	89(10.5)	23(11.0)	
Estimated fetal weight at TOLAC delivery (g)	2995±480	3145±375	<0.001
Lower uterine segment thickness (mm)	2.64±0.67	2.68±0.67	0.672
Cervical dilatation on admission At TOLAC delivery (5~6cm)	139(21.0)	19(12.4)	0.001
Cervical effacement on admission At TOLAC delivery (80~100%)	543(81.5)	106(69.3)	0.001
Cervical score	8.1±2.8	7.5±2.5	0.016
Pre-existing medical condition			
GDM	166(19.1)	46(21.7)	0.398
Preeclampsia	24(2.8)	6(2.8)	0.959

GDM, gestational diabetes mellitus.

Data are expressed as mean ± standard deviation or n (%).
